# Integrative bioinformatics analysis of miRNA and mRNA expression profiles and identification of associated miRNA-mRNA network in intracranial aneurysms

**DOI:** 10.1016/j.ncrna.2024.01.004

**Published:** 2024-01-09

**Authors:** Dongxiao Xu, Ilgiz Gareev, Ozal Beylerli, Valentin Pavlov, Huang Le, Huaizhang Shi

**Affiliations:** a Department of Neurosurgery, The First Affiliated Hospital of Henan University of Science and Technology, Luoyang, China; b Central Research Laboratory, Bashkir State Medical University, Ufa, Republic of Bashkortostan, 3 Lenin street, 450008, Russia; c Department of Neurosurgery, The First Affiliated Hospital of Harbin Medical University, Harbin, 150001, China; d Department of Urology, Bashkir State Medical University, 3 Lenin Street, 450008, Ufa, Russia

**Keywords:** Intracranial aneurysms, Aortic aneurysm, Bioinformatical analysis, Transcription factors, Regulatory network, miRNA, mRNA

## Abstract

**Background:**

Intracranial aneurysms (IAs) represent protrusions in the vascular wall, with their growth and wall thinning influenced by various factors. These processes can culminate in the rupture of the aneurysm, leading to subarachnoid hemorrhage (SAH). Unfortunately, over half of the patients prove unable to withstand SAH, succumbing to adverse outcomes despite intensive therapeutic interventions, even in premier medical facilities. This study seeks to discern the pivotal microRNAs (miRNAs) and genes associated with the formation and progression of IAs.

**Methods:**

The investigation gathered expression data of miRNAs (from GSE66240) and mRNAs (from GSE158558) within human aneurysm tissue and superficial temporal artery (STA) samples, categorizing them into IA and normal groups. This classification was based on the Gene Expression Omnibus (GEO) database.

**Results:**

A total of 70 differentially expressed microRNAs (DEMs) and 815 differentially expressed mRNAs (DEGs) were pinpointed concerning IA. Subsequently, a miRNA-mRNA network was constructed, incorporating 9 significantly upregulated DEMs and 211 significantly downregulated DEGs. Simultaneously, functional enrichment and pathway analyses were conducted on both DEMs and DEGs. Through protein-protein interaction (PPI) network analysis and functional enrichment, 9 significantly upregulated DEMs (hsa-miR-188-5p, hsa-miR-590-5p, hsa-miR-320b, hsa-miR-423-5p, hsa-miR-140-5p, hsa-miR-486-5p, hsa-miR-320a, hsa-miR-342-3p, and hsa-miR-532-5p) and 50 key genes (such as ATP6V1G1, KBTBD6, VIM, PA2G4, DYNLL1, METTL21A, MDH2, etc.) were identified, suggesting their potential significant role in IA. Among these genes, ten were notably negatively regulated by at least two key miRNAs.

**Conclusions:**

The findings of this study provide valuable insights into the potential pathogenic mechanisms underlying IA by elucidating a miRNA-mRNA network. This comprehensive approach sheds light on the intricate interplay between miRNAs and genes, offering a deeper understanding of the molecular dynamics involved in IA development and progression.

## 1 Introduction

Intracranial aneurysms (IAs) represent pathological localized protrusions of the arterial wall, exhibiting a distinct deviation from the typical three-layer structure of the vascular wall. The rupture of IAs stands as the predominant cause, accounting for approximately 85 % of non-traumatic subarachnoid hemorrhage (SAH). This rupture results in the infiltration of blood into the subarachnoid space of the brain, contributing to non-traumatic intracerebral hemorrhage (ICH), a phenomenon more prevalent in individuals of working age [1,2]. The intricate progression of aneurysmal disease carries the potential for severe and enduring neurological deficits or even mortality. Various factors are believed to be associated with the occurrence of IAs, encompassing age, hypertension, genetic predisposition, hemodynamic alterations, and environmental influences [3]. Despite numerous studies on IAs, the precise pathophysiological mechanisms governing the formation, progression, and rupture of aneurysms remain elusive. The complex interplay of these factors and their contribution to the pathological evolution of IAs poses a significant challenge in comprehending the intricate dynamics of aneurysm development. Consequently, unveiling the underlying mechanisms holds crucial importance for advancing our understanding of these vascular abnormalities and facilitating the development of targeted interventions for their prevention and treatment.

MicroRNAs (miRNAs), small non-coding RNA molecules spanning approximately 18–22 nucleotides, serve as potent post-transcriptional regulators of gene expression. Their regulatory process is manifested through the binding to the 3′-untranslated (3′-UTR) regions of target mRNAs, specifically those encoding protein-coding genes, thereby exerting a pronounced negative impact on their translation processes [4]. The indisputable significance of miRNAs in diverse biological phenomena, including cell cycle control, proliferation, differentiation, and apoptosis, underscores their pivotal role in orchestrating intricate cellular functions [4]. The expanding body of evidence compellingly establishes the pivotal role of miRNAs in the initiation, growth, and progression of IAs [5,6]. The multifaceted pathophysiology characterizing IAs, marked by endothelial dysfunction, phenotypic modulation of vascular smooth muscle cells (VSMCs), and the accumulation of inflammatory cells, is intricately governed by the regulatory influence of miRNAs [5]. Yet, a critical knowledge gap persists, necessitating a comprehensive exploration to identify the specific miRNAs and their target genes implicated in these intricate IA-associated processes. This study aspires to unravel the fundamental pathophysiological pathways integral to the molecular mechanisms dictating the formation and development of IAs. By leveraging miRNA and mRNA expression profiles, our aim is to pinpoint and elucidate the pivotal players within these pathways. Furthermore, we endeavor to construct a comprehensive miRNA-mRNA regulatory network, offering nuanced insights into the intricate dynamics underpinning IA formation and development. Envisioning that these findings will not only enhance our understanding of IA pathogenesis but also pave the way for the development of sophisticated diagnostic tools and molecularly targeted therapies, we are poised to unlock novel avenues for addressing the complex challenges posed by IAs in clinical settings.

## 2 Materials and methods

### 2.1 Microarray data

**Fig. 1:**
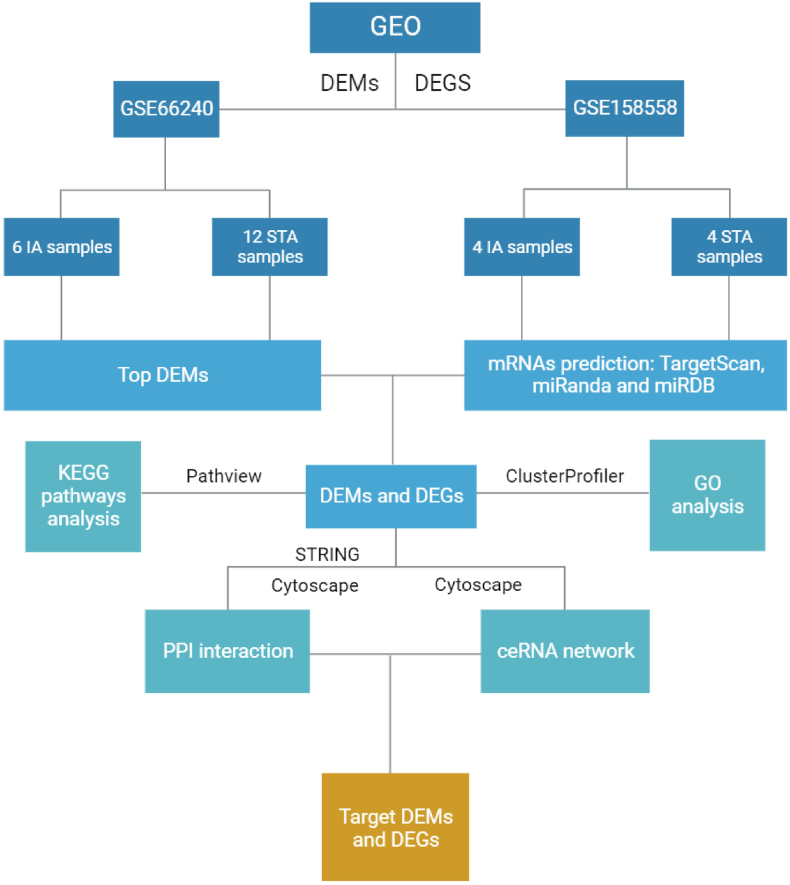
The workflow of the study.

The microarray datasets containing miRNA and mRNA expression profiles linked to IAs were acquired from the Gene Expression Omnibus (GEO) database, administered by the National Center for Biotechnology Information (NCBI), and can be accessed at https://www.ncbi.nlm.nih.gov/geo/); accessed in September 2022). Notably, the miRNA expression dataset, GSE66240, was conducted on the GPL17303 platform, while the mRNA expression dataset, GSE158558, utilized the GPL20301 platform (Illumina HiSeq 4000). This dataset encompasses a total of 26 samples, comprising 10 samples derived from individuals with IAs and 16 from specimens of the superficial temporal artery (STA). To ensure methodological consistency and precision across various datasets and platforms, distinctive preprocessing techniques were applied. Specifically, the miRNA microarray analysis employed the miRCURY LNA Array (version 11.0; Exiqon, Vedbaek, Denmark). Meanwhile, for the mRNA microarray, the oligo package in R (version 3.4.2) was employed. Rigorous procedures for background correction and normalization were implemented on raw data, ensuring the reliability and robustness of subsequent analyses. Crucially, it is essential to underscore that ethical committee approval was considered unnecessary for this study. This decision is well-founded on the premise that the datasets utilized were sourced from public databases and were handled with strict adherence to GEO publication guidelines and data access policies. Consequently, the study aligns with established ethical standards for handling publicly available datasets, reflecting a commitment to responsible and ethical data utilization principles. This approach emphasizes transparency and integrity in the utilization of data from public repositories, eliminating the need for specific ethical clearances in this context. Fig. 1 demonstrates the workflow of the study.

### 2.2 Identification of DEGs

Differentially expressed genes (DEGs) distinguishing between IA and STA samples were identified through the utilization of GEO2R, an interactive web tool accessible at http://www.ncbi.nlm.nih.gov/geo/geo2r. GEO2R facilitates the comparative analysis of two or more datasets within a GEO series, enabling the identification of DEGs across various experimental conditions. In this analysis, P-values, along with the Benjamini and Hochberg false discovery rates, were employed to strike a balance between pinpointing statistically significant genes and mitigating the risk of false positives. To enhance the robustness of the results, several data refinement steps were implemented. Probe sets lacking corresponding gene symbols were excluded, and in cases where genes were represented by more than one probe set, either the removal or averaging of such duplicates was undertaken. A stringent statistical criterion was applied, considering a Fold Change (LogFC) greater than 1.5 and a P-value less than 0.05 as indicative of statistical significance. These parameters were chosen to ensure the identification of DEGs that not only reached statistical significance but also exhibited a biologically meaningful degree of differential expression.

### 2.3 Enrichment analysis

The Gene Ontology (GO) database serves as a valuable resource for predicting the functional annotations of gene products across categories such as GO-biological process (BP), GO-cellular component (CC), and GO-molecular function (MF) [7]. Simultaneously, the Kyoto Encyclopedia of Genes and Genomes (KEGG) database is commonly employed to anticipate the pathways involving specific genes [8]. To decipher the functional implications of differentially expressed microRNAs (DEMs) and their negatively correlated target genes, the clusterProfiler package (version 3.8.1; accessible at http://www.bioconductor.org/packages/release/bioc/html/clusterProfiler.html) [9] was utilized. Through the clusterProfiler package, enrichment analyses for GO and KEGG were conducted on the identified DEMs, shedding light on the biological processes, cellular components, and molecular functions implicated in the context of these miRNA expressions. Subsequently, the genes within this module underwent further GO and KEGG analyses using the DAVID tool. A false discovery rate (FDR) threshold of <0.05 was defined as significant, ensuring a stringent criterion for the identification of biologically meaningful enrichments. This integrative approach provides a comprehensive understanding of the functional landscape associated with the identified miRNAs and their target genes, enhancing our insights into the intricate molecular mechanisms at play in the context of IAs.

### 2.4 PPI network analysis

The prediction of the protein–protein interaction (PPI) network was carried out using the Retrieval of Interacting Genes (STRING) online database search tool, accessible at http://string-db.org (version 10.0) [10]. Analyzing functional interactions among proteins is instrumental in gaining insights into the mechanisms underlying disease occurrence and development. In this study, the PPI network for DEGs was constructed utilizing the STRING database, with interactions possessing a composite score greater than 0.4 considered statistically significant. Cytoscape, an open-source bioinformatics software platform for visualizing molecular interaction networks (version 3.4.0) [11], was employed to construct the PPI networks. Further refinement and identification of crucial modules within the PPI networks were facilitated by the Molecular Complex Discovery (MCODE) plug-in (version 1.4.2) [12]. MCODE is an application designed for clustering networks based on topology, aiding in the identification of densely connected regions. In this context, the PPI networks were created using Cytoscape, and the most significant module was pinpointed using MCODE. The selection criteria for identifying significant modules were set as follows: MCODE scores exceeding 5, a degree cutoff of 2, a node score cutoff of 0.2, a maximum depth of 100, and a k-score of 2. This approach ensures a comprehensive exploration of the PPI network, emphasizing the detection of densely connected regions that may play pivotal roles in the context of the identified DEGs related to IAs.

### 2.5 Construction of the miRNA-targeted gene network

The identification of downstream target genes based on the Differentially Expressed MicroRNAs (DEMs) derived from the analysis of datasets GSE66240 and GSE50867 involved a multi-step process. Utilizing the TargetScan (http://www.targetscan.org/vert_71/), miRanda (http://zmf.umm.uni-heidelberg.de/apps/zmf/mirwalk), and miRDB (http://www.mirdb.org/) databases, predictions of downstream target genes were performed. The resulting target genes from these three databases were then compared with the dataset [[13], [14], [15]]. Simultaneously, the DEMs from the GSE158558 dataset were considered, and an intersection was taken to identify candidate target genes. Leveraging the regulatory relationships between miRNAs and mRNAs, a comprehensive miRNA-mRNA regulatory network was established. To further enhance the regulatory network's complexity, predictions of Transcription Factors (TFs) that regulate miRNAs were made using the Transcriptional Regulatory Relationships Unraveled by Sentence-based Text mining (TRRUST) database, accessible at http://www.grnpedia.org/trrust/ [16]. This approach relies on existing literature to predict TFs influencing miRNA regulation. By incorporating data from multiple sources and databases, this comprehensive strategy ensures a thorough exploration of the regulatory landscape, unveiling potential interactions and providing a nuanced understanding of the intricate relationships between miRNAs, mRNAs, and transcription factors.

## 3 Results

### 3.1 Differential expression analysis

**Fig. 2:**
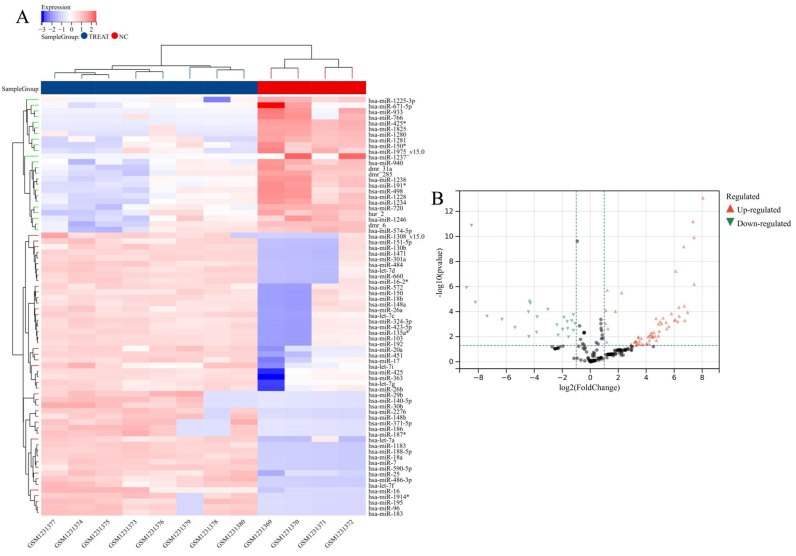
Illustrates the expression profiles and cluster analysis of microRNAs (miRNAs) in intracranial aneurysm (IA) samples. In panel (A), a heatmap showcases the differentially expressed microRNAs (DEMs). The color scheme employs blue for IA samples and red for control samples, effectively portraying the distinct expression patterns between the two groups. In panel (B), a volcano plot represents the DEMs, emphasizing the relationship between statistical significance and fold change. Red dots signify upregulation, green dots denote downregulation, and gray dots indicate no significant differential expression. This visualization offers a clear and concise representation of the magnitude and significance of expression changes in the analyzed miRNAs.

**Fig. 3:**
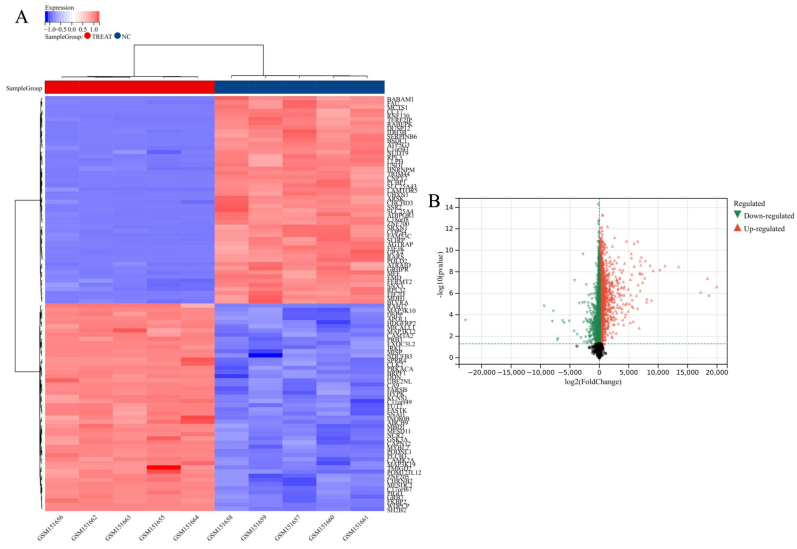
Presents the expression profiles and cluster analysis of gene targets in intracranial aneurysm (IA) samples. In panel (A), a heatmap illustrates the expression values of differentially expressed genes (DEGs). The color scheme assigns red to IA samples and blue to control samples, effectively depicting the distinctive expression patterns between the two groups. In panel (B), a volcano plot showcases the DEGs, emphasizing the relationship between statistical significance and fold change. Red dots indicate upregulation, green dots signify downregulation, and gray dots denote no significant differential expression. This visual representation succinctly communicates the magnitude and significance of expression changes in the analyzed genes.

A thorough examination of the GSE66240 dataset revealed a total of 70 Differentially Expressed MicroRNAs (DEMs), providing insights into the distinctive expression patterns between samples associated with IA and their normal counterparts. Within this set, 46 DEMs displayed upregulation, while 24 DEMs exhibited downregulation, as illustrated in Fig. 2A–B. Noteworthy among these were specific miRNAs, such as hsa-miR-188-5p, hsa-miR-590-5p, hsa-miR-320b, hsa-miR-423-5p, hsa-miR-140-5p, hsa-miR-486-5p, hsa-miR-320a, hsa-miR-342-3p, and hsa-miR-532-5p, which consistently demonstrated expression patterns across both datasets. Particularly, hsa-miR-188-5p and hsa-miR-590-5p consistently exhibited high expression levels, characterized by log2FC values exceeding 2 in both datasets. Visual representations of these DEGs through heatmaps, subjected to hierarchical cluster analysis (Fig. 3A–B), underscored their distinctive expression patterns between IA and normal groups. In a comprehensive computational analysis, target genes associated with DEMs were identified through predictions from TargetScan, miRanda, and miRDB. This approach resulted in the identification of 615 downregulated genes (Table 1). The integration of these findings not only accentuates the robustness of the identified DEMs but also provides valuable insights into potential downstream target genes, thereby enhancing our understanding of their pivotal roles in the context of IAs.

**Table 1:** Key microRNAs (miRNAs) that are differentially expressed in intracranial aneurysm (IA) and their respective target genes.

miRNAs	Fold change [Log(Fold Change)]	P-value	Target gene (number of total target genes)
hsa-miR-188-5p	8.054578338	0.0084393	C9orf72; CTNNBIP1; AKAP2; ARPC2; CCNT2; BAG5; C6orf106; CBFB; CCDC6; CNIH1; CCNG1; CD2AP; CPNE8; ATP6V1G1; etc. (54)
hsa-miR-590-5p	6.685881638	0.0028131	AIM1L; ALX1; AP1AR; ARHGAP24; ARMCX1; ASF1A; BCL7A; BEST3; BRWD1; C10orf12; CASKIN1; CCL1; CCL22; CD69; CHIC1; CPEB3; DMRTC1B; DUSP8; ELF2; ERG; FAM13A; FASLG; FGF18; GLCCI1; GLIS2; GRAMD3; IL12A; etc. (84)
hsa-miR-320b	0.76350005	0.0088756	COPS2; ALKBH5; FOXQ1; CNKSR2; ARFIP1; ARL8B; ARMCX2; ARPP19; BCAP29; BANP; CD274; CDH20; DAZAP1; DBN1; DESI2; DHX15; DLX1; ASH2L; EFS; EIF2B1; DPY30; DDX42; EMC7; EOGT; ETFA; CCR7; FTL; CDK13; etc. (95)
hsa-miR-423-5p	4.321941388	0.0099192	AGAP2; AMBRA1; ANGPTL2; AP2M1; ASB14; ASB6; ATP8B2; ATXN7L3; BAP1; BCAS3; C20orf27; CDC42SE1; CNBP; CNTN2; CREBZF; CRTC2; CYP4F22; DERL3; DOLPP1; EFNA3; EIF4A1; etc. (106)
hsa-miR-140-5p	4.457337875	0.0039776	AARS; ADAM10; ADAM9; ADAMTS5; AFTPH; AKAP2; AKIRIN2; AMER2; ANKFY1; ANKRD12; ANO6; AP2B1; ARHGAP19; ARL15; BACH1; BAG2; BCL2L1; BMP2; C1R; C6orf47; CALU; CAMK2N1; CAND1; CAPN1; CCNYL1; CELF1; CELF2; CEP63; CERCAM; CORO2A; CREB3L1; etc. (203)
hsa-miR-486-5p	1.165140625	0.0019387	C5orf64; BTAF1; BTBD3; ARMC8; ARHGAP5; ABHD17A; AFF3; ARHGAP44; ARID4B; ASB4; ATXN7L3; CELF2; CDH7; COL6A6; ABHD17B; etc. (59)
hsa-miR-320a	3.157015113	0.0099146	DAZAP1; ARFIP1; GRASP; DHX15; CCR7; CD274; CDH20; FUS; ARL8B; ARMCX2; ARPP19; ASH2L; BANP; CDK13; CNKSR2; COPS2; DPY30; EFS; EIF2B1; EMC7; DBN1; DDX42; DESI2; DLX1; EOGT; ETFA; FOXQ1; FTL; GNAI1; BCAP29; etc. (94)
hsa-miR-342-3p	3.471722613	0.0035678	AFP; AKIRIN1; AP2M1; BCL2L1; BTN2A1; C10orf35; CA12; CAMKV; CASP2; CTBP2; DDX50; DTNBP1; EP300; EPC1; FAM208A; FAM53C; FOXQ1; etc. (51)
hsa-miR-532-5p	3.31352065	0.0036788	APBB2; ATP2C1; BHLHB9; C11orf31; CAPN3; CCDC64; CCNG1; CCR4; CD40LG; CPEB3; CRIPT; CXCL1; CXCL2; CYCS; DDHD1; DENND6A; DHFR; ERCC6L; etc. (69)

### 3.2 Protein–protein interaction network analysis

**Fig. 4:**
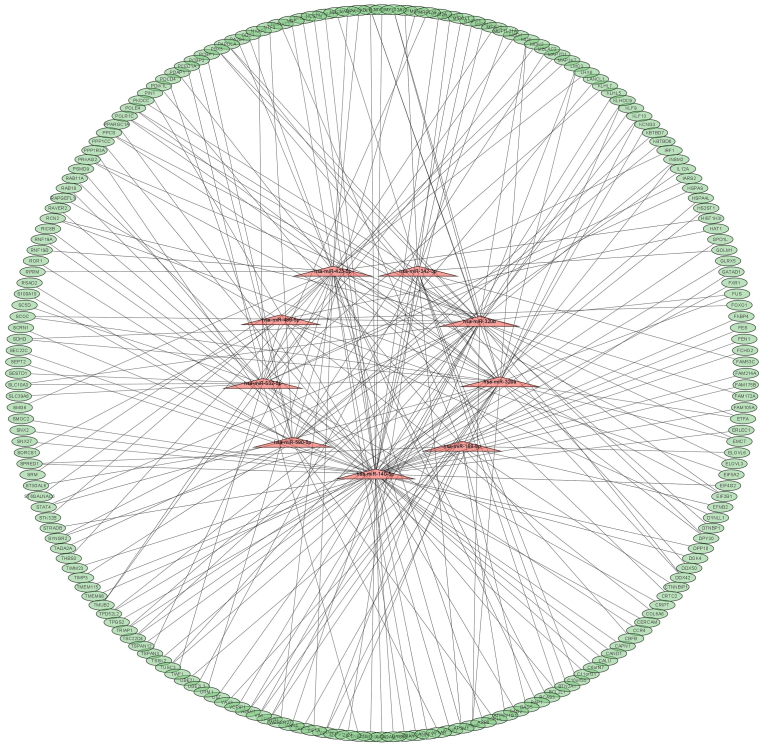
Illustrates the construction of the protein-protein interaction (PPI) network, showcasing the interconnected relationships between downregulated differentially expressed genes (DEGs) and the targeted 9 upregulated differentially expressed microRNAs (DEMs). In this network, nodes represent individual genes, and the lines connecting them depict the interactions between these genes. This visual representation offers insights into the complex web of interactions between the identified genes and microRNAs, providing a comprehensive overview of their regulatory relationships in the context of intracranial aneurysms (IAs).

A miRNA-mRNA regulatory network was constructed to illustrate the interactions between 9 upregulated miRNAs and their targeted mRNAs. Fig. 4 and Table 2 visually represent this network, showcasing the regulatory relationships between each miRNA and the corresponding mRNAs.

**Table 2:** The predominant upregulated differentially expressed microRNAs (DEMs) identified in intracranial aneurysm (IA) and the corresponding significantly differentially expressed genes (DEGs).

miRNAs	Target genes
hsa-miR-188-5p	ATP6V1G1; BAG5; CBFB; CTNNBIP1; EFNB2; ELOVL6; GATAD1; KCNG3; NMNAT3; PDIK1L; SPRED1; UBE2I; VAV3; XRCC5; ZFP91
hsa-miR-590-5p	KBTBD6; TIMP3; PCBP1; PCBP2; S100A10; LANCL1; ASF1A; AP1AR; ST3GAL6; RSAD2; PDCD4; NTF3; KBTBD7; MSH2; MBLAC2; IL12A; PPP1R3A; TADA2A; VIM
hsa-miR-320b	VIM; MYL12A; YWHAH; TSC22D4; TPD52L2; FUS; SCOC; ARL8B; VPS37B; POLE4; RCN2; PAPOLA; EMC7; KLF13; NAA20; DDX42; DPY30; SDHD; VDAC1; EIF2B1; PPCS; MDK; ETFA; LMO3; POLR1C; PBX3; TRIAP1; STAT4; SYNGR2; PRKAG2; SLC10A3; KLHDC9; TUSC3; INSM2
hsa-miR-423-5p	PA2G4; ODC1; SRM; MAP7D1; ANGPTL2; TSPAN3; AP2M1; PDAP1; MFAP2; YIF1A; FAM172A; CRTC2; THBS3; AMBRA1; URM1; NGF; BCAS3; TMUB2; RPRM; BAP1; RNF19B; HIST1H3I; STK32B; ELOVL3; NAALADL1; LHX6; SMG6; WBSCR27; NAGPA; ASB6
hsa-miR-140-5p	KLF9; SEPT2; CALU; NCKAP1; EIF4G2; AARS; PIN1; GLRX5; RAB11A; SMOC2; NFE2L2; TMEM98; SNX2; PPP1CC; ROR1; BAG2; FXR1; ERLEC1; FCHO2; ADAMTS5; SPRED1; TMEM115; RIC8B; CAPN1; NAA20; FKBP4; MYO10; RNF19A; NCSTN; SCRN1; SNX27; CAND1; C6orf47; ANKFY1; GPD1L; TIMM23; KLHL5; ZNF800; MICAL3; AP2B1; UST; FAM175B; PPARGC1A; SEC22C; HSPA4L; BCL2L1; HS2ST1; TPGS2; CERCAM; FES; MINPP1; STRADB; MOB3A; FAM214A; FAM105A; DOK4; VCPIP1; TSPAN12; MIPOL1; WNK4; FEN1; TSSK2; DPP10
hsa-miR-486-5p	DYNLL1; TWF1; FOXO1; ST6GALNAC6; HAT1; EIF5A2; MAP3K7; SORCS1; RAPGEFL1; COL6A6
hsa-miR-320a	VIM; MYL12A; YWHAH; TSC22D4; TPD52L2; FUS; SCOC; ARL8B; VPS37B; POLE4; RCN2; PAPOLA; EMC7; KLF13; NAA20; DDX42; DPY30; SDHD; VDAC1; EIF2B1; PPCS; MDK; ETFA; LMO3; POLR1C; PBX3; TRIAP1; STAT4; SYNGR2; PRKAG2; SLC10A3; KLHDC9; TUSC3; INSM2
hsa-miR-342-3p	METTL21A; MTDH; DDX50; PKDCC; UBE2L3; FAM53C; MMS19; AP2M1; IRF1; BTN2A1; C10orf35; BCL2L1; DTNBP1; NBEA; ZIC4; GOLM1
hsa-miR-532-5p	MDH2; HSPA9; C11orf31; RAB18; SESTD1; MRPL18; PCED1A; IARS2; SC5D; CRIPT; MED1; TMUB2; RAVER2; ZNF250; KLHL7; CCR4; PSMD9; SLC39A8; NYAP2

**Fig. 5:**
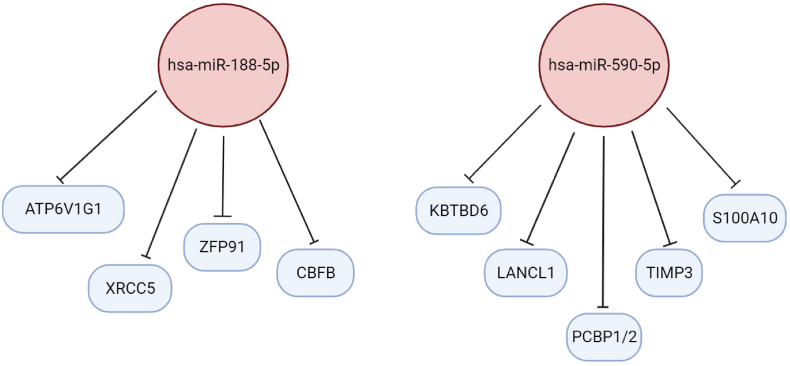
Red nodes signify a strong expression level of 2 top microRNAs (miRNAs), while blue nodes signify a low level of expression levels of top their target genes.

Specifically, hsa-miR-188-5p exhibited significant negative regulation on 15 targeted mRNAs, hsa-miR-590-5p negatively regulated 19 targeted mRNAs, hsa-miR-320b negatively regulated 34 targeted mRNAs, hsa-miR-423-5p negatively regulated 30 targeted mRNAs, hsa-miR-140-5p negatively regulated 63 targeted mRNAs, hsa-miR-486-5p negatively regulated 10 targeted mRNAs, hsa-miR-320a negatively regulated 34 targeted mRNAs, hsa-miR-342-3p negatively regulated 16 targeted mRNAs, and hsa-miR-532-5p negatively regulated 19 targeted mRNAs. The results were retrieved and visualized using Cytoscape. Among the 221 significantly downregulated Differentially Expressed Genes (DEGs), the 10 most significant genes were identified as ATPase H+ transporting V1 subunit G1 (ATP6V1G1), X-ray repair cross-complementing protein 5 (XRCC5), ubiquitination enzyme zinc finger protein 91 (ZFP91), core binding factor beta (CBFB), kelch repeat and BTB domain-containing 6 (KBTBD6), tissue inhibitor of metalloproteinase 3 (TIMP3), polyC-RNA-binding protein 1 (PCBP1), PolyC-RNA-binding protein 2 (PCBP2), S100 calcium-binding protein A10 (S100A10), and Lanthionine synthase C-like protein-1 (LANCL1). Notably, hsa-miR-188-5p and hsa-miR-590-5p exhibited the most extensive connections with these genes (Table 3) (Fig. 5). Additionally, it is worth mentioning that within the hsa-miR-320 family, specifically hsa-miR-320a and hsa-miR-320b, common target genes were identified. However, hsa-miR-320a demonstrated higher expression levels compared to hsa-miR-320b. This intricate regulatory network provides a comprehensive view of the interactions between upregulated miRNAs and their downstream target genes, shedding light on potential key players in the context of IAs.

**Table 3:** Nine upregulated differentially expressed microRNAs (DEMs) in intracranial aneurysm (IA) and their 50 most significant target genes.

miRNAs	miRNAs fold change [Log(Fold Change)]	Top target genes	mRNAs fold change [Log(Fold Change)]
hsa-miR-188-5p	8.054578338	ATP6V1G1	−887.349197
		XRCC5	−543.611825
		ZFP91	−305.6641354
		CBFB	−258.4137301
hsa-miR-590-5p	6.685881638	KBTBD6	−3969.043051
		TIMP3	−2380.258042
		PCBP1	−1050.75371
		PCBP2	−380.9329573
		S100A10	−330.6388879
		LANCL1	−218.8268424
hsa-miR-320b	0.76350005	VIM	−5568.64642
		MYL12A	−2126.928036
		YWHAH	−1303.165809
		TSC22D4	−408.7456301
		TPD52L2	−354.020199
		FUS	−332.519518
		SCOC	−254.1273705
		ARL8B	−251.0638471
		VPS37B	−202.5026557
hsa-miR-423-5p	4.321941388	PA2G4	−263.0269861
		ODC1	−206.1051254
hsa-miR-140-5p	4.457337875	KLF9	−1610.98743
		SEPT2	−1345.744109
		CALU	−1035.291506
		NCKAP1	−768.1398445
		EIF4G2	−765.7836147
		AARS	−534.2845671
		PIN1	−450.5483592
		GLRX5	−269.0178919
		RAB11A	−261.315547
		SMOC2	−257.8299924
		NFE2L2	−253.5985164
		TMEM98	−245.0455623
		SNX2	−233.1927743
		PPP1CC	−224.7559959
		ROR1	−210.6931393
hsa-miR-486-5p	1.165140625	DYNLL1	−674.1210323
		TWF1	−217.8284361
hsa-miR-320a	3.157015113	VIM	−5568.64642
		MYL12A	−2126.928036
		YWHAH	−1303.165809
		TSC22D4	−408.7456301
		TPD52L2	−354.020199
		FUS	−332.519518
		SCOC	−254.1273705
		ARL8B	−251.0638471
		VPS37B	−202.5026557
hsa-miR-342-3p	3.471722613	METTL21A	−263.066903
hsa-miR-532-5p	3.31352065	MDH2	−595.5935079
		HSPA9	−331.4200711

### 3.3 Enrichment analysis of DEMs and DEGs

**Fig. 6:**
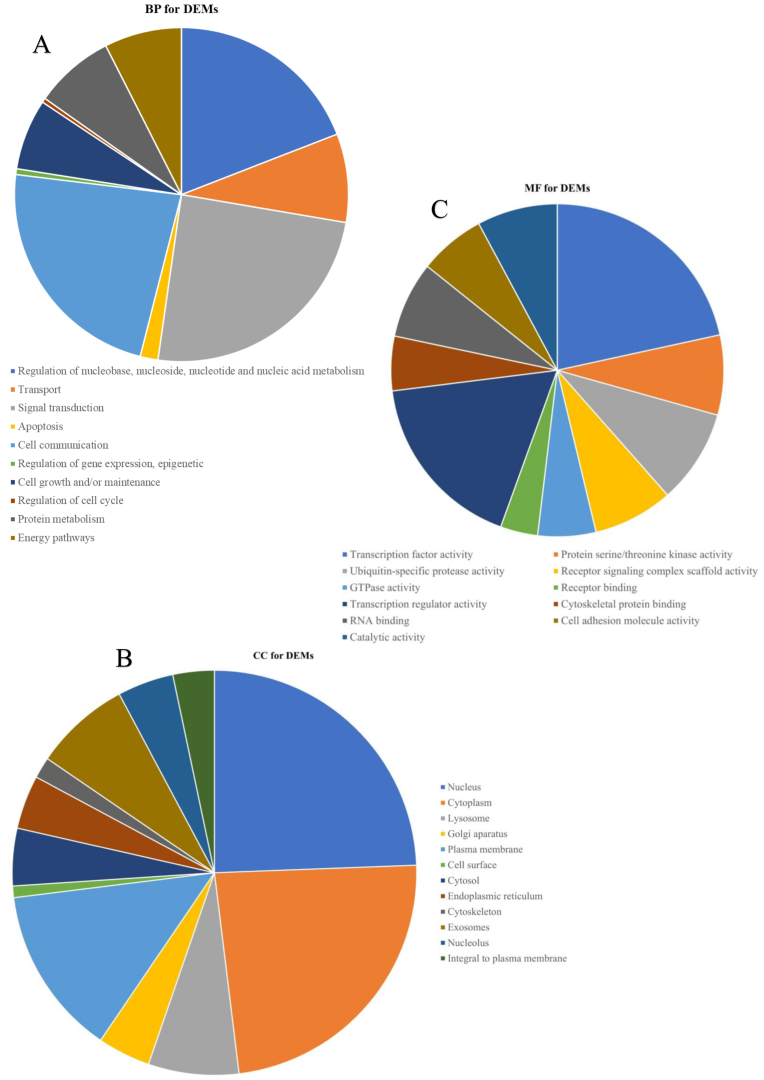
Depicts the outcomes of Gene Ontology (GO) functional enrichment analysis for the identified differentially expressed microRNAs (DEMs). In panel (A), the Biological Process (BP) category highlights the involvement of DEMs in processes such as the regulation of nucleobase, nucleoside, nucleotide, and nucleic acid metabolism, as well as functions related to transport and signal transduction. Panel (B) illustrates the Cellular Component (CC) category, showcasing enrichments in various cellular locales, including the nucleus, cytoplasm, lysosome, Golgi apparatus, and exosomes. Finally, in panel (C), the Molecular Function (MF) category outlines enrichments in activities such as transcription factor activity, protein serine/threonine kinase activity, ubiquitin-specific protease activity, and transcription regulator activity. These visual representations provide a detailed insight into the diverse functional characteristics associated with the identified DEMs.

**Fig. 7:**
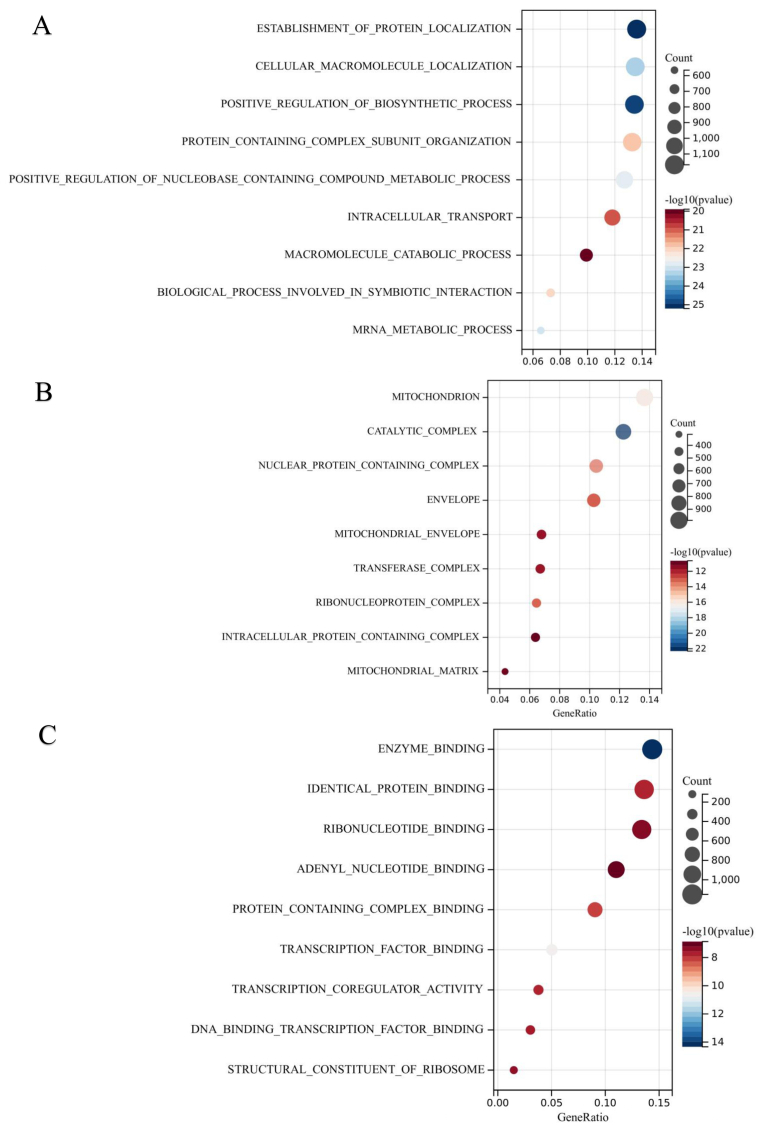
Illustrates the outcomes of Gene Ontology (GO) enrichment analysis for the candidate target genes. Panel (A) focuses on GO-biological process (BP), highlighting enrichments in processes such as the establishment of protein localization, cellular macromolecule localization, positive regulation of biosynthetic processes, and the organization of protein-containing complex subunits. In Panel (B), GO-cellular component (CC) terms reveal enrichments in cellular structures, including mitochondria, catalytic complexes, nuclear protein-containing complexes, and the envelope. Lastly, panel (C) delineates GO-molecular function (MF) terms, showcasing enrichments in activities such as enzyme binding, identical protein binding, ribonucleotide binding, and adenyl nucleotide binding. These visual representations provide a detailed overview of the functional characteristics associated with the candidate target genes.

**Fig. 8:**
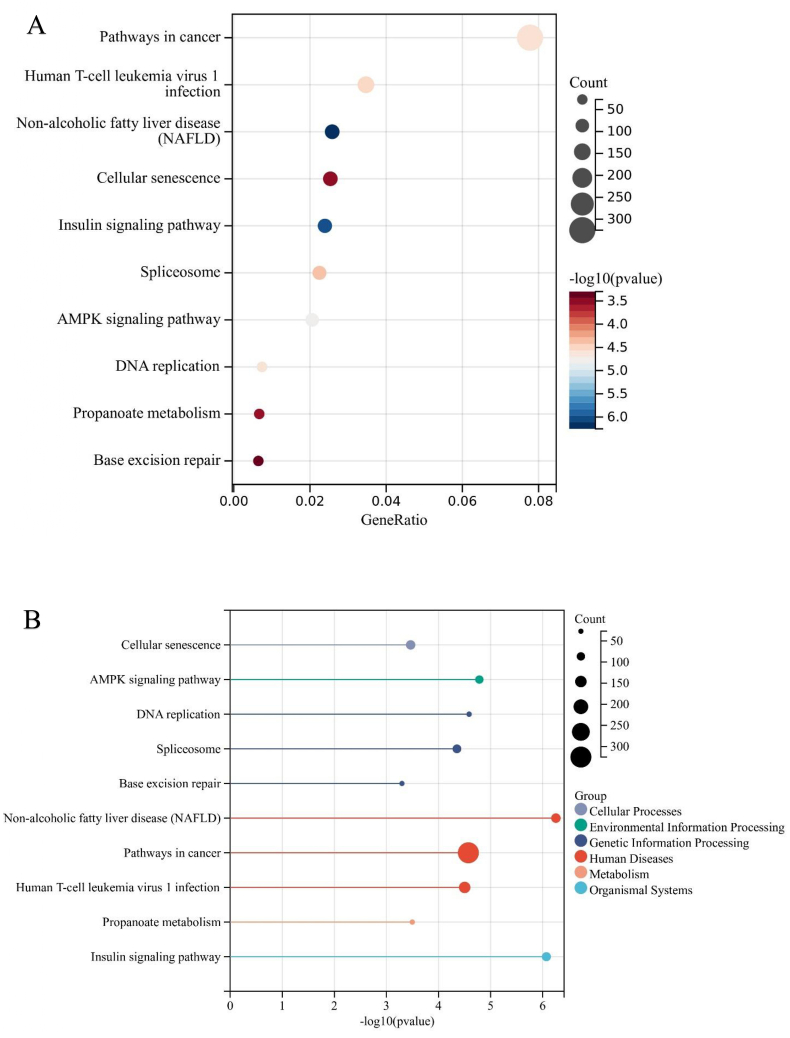
Presents the outcomes of the Kyoto Encyclopedia of Genes and Genomes (KEGG) pathway analysis conducted for the candidate target genes. In Panel (A), a KEGG Bar plot outlines the distribution of differentially expressed genes (DEGs) across various pathways. Panel (B) presents a KEGG Bubble plot, visually representing the significance and magnitude of enrichment for DEGs within different KEGG pathways. These visualizations collectively provide insights into the pathways associated with the candidate target genes.

To comprehensively explore the biological functions attributed to the upregulated DEMs, an extensive analysis of GO pathway enrichment was conducted. The findings unveiled notable enrichment across a spectrum of functional characteristics. In terms of BP, the DEMs exhibited substantial involvement in processes encompassing the regulation of nucleobase, nucleoside, nucleotide, and nucleic acid metabolism, as well as functions related to transport and signal transduction, among others (Fig. 6A). Pertaining to CC, enrichments were observed in diverse cellular locales, including the nucleus, cytoplasm, lysosome, Golgi apparatus, exosomes, and various other cellular components (Fig. 6B). Within the domain of MF, the DEMs displayed enrichments in activities such as transcription factor activity, protein serine/threonine kinase activity, ubiquitin-specific protease activity, transcription regulator activity, and more (Fig. 6C). Moreover, GO and KEGG pathway enrichment analyses were systematically conducted for the DEGs identified within these modules. In terms of BP, the DEGs were predominantly associated with processes involving the establishment of protein localization, cellular macromolecule localization, positive regulation of biosynthetic processes, and the organization of protein-containing complex subunits (Fig. 7A). Regarding CC, enrichments were discerned in various cellular structures, including mitochondria, catalytic complexes, nuclear protein-containing complexes, the envelope, and more (Fig. 7B). MF enrichments encompassed activities such as enzyme binding, identical protein binding, ribonucleotide binding, adenyl nucleotide binding, and other functions (Fig. 7C). In the subsequent KEGG pathway enrichment analysis, the DEGs displayed significant enrichment in pathways associated with cellular senescence, the AMP-activated protein kinase (AMPK) signaling pathway, DNA replication, the spliceosome, base excision repair, non-alcoholic fatty liver disease (NAFLD), pathways in cancer, human T-cell leukemia virus 1 infection, propanoate metabolism, and insulin (Fig. 8A–B). This comprehensive analysis provides a nuanced understanding of the diverse biological processes and pathways linked to the identified DEMs and DEGs, contributing valuable insights into their potential roles in the context of IAs.

### 3.4 Screening of potential transcription factors

**Fig. 9:**
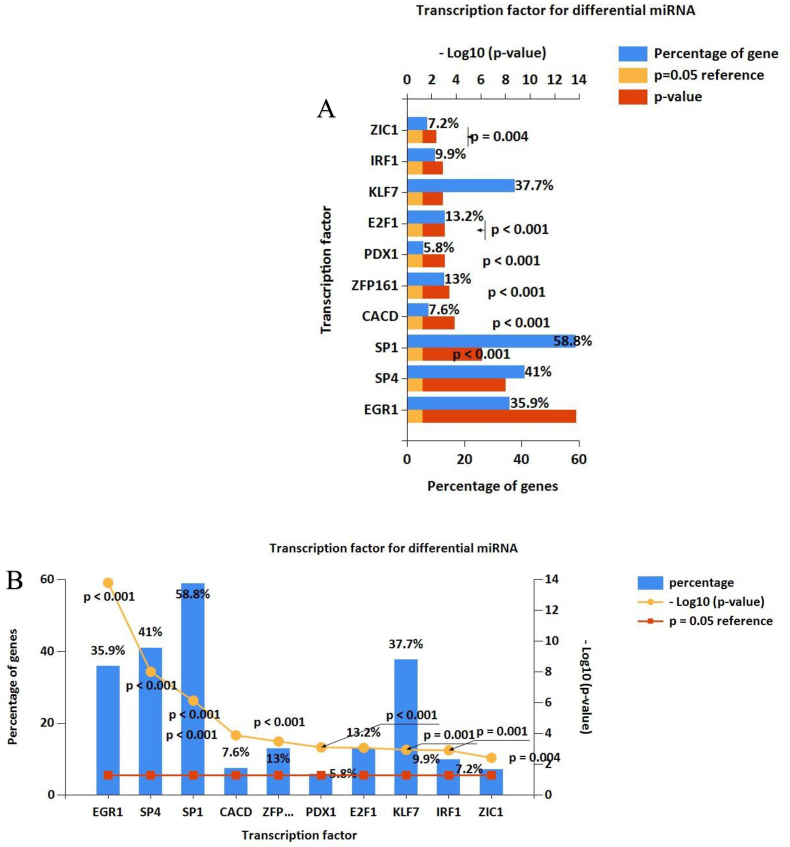
Depicts the enriched transcription factors (TFs) identified through the analysis of target genes associated with differentially expressed microRNAs (DEMs). The top 10 most significant TFs include Zic family member 1 (ZIC1), Interferon regulatory factor 1 (IRF1), Krueppel-like factor 7 (KLF7), E2F transcription factor 1 (E2F1), Pancreatic duodenal homeobox 1 (PDX1), ZFP161, CACD, Specificity protein 1 (SP1), Specificity protein 4 (SP4), and Early growth response protein 1 (EGR1) (Fig. 7A–B).

In this study, the identification of the most significant TFs for miRNAs resulted in the recognition of the top 10 TFs. These included Zic family member 1 (ZIC1), Interferon regulatory factor 1 (IRF1), Krueppel-like factor 7 (KLF7), E2F transcription factor 1 (E2F1), Pancreatic duodenal homeobox 1 (PDX1), ZFP161, CACD, Specificity protein 1 (SP1), Specificity protein 4 (SP4), and Early growth response protein 1 (EGR1) (Fig. 9A–B).

## 4 Discussion

IAs are localized pathological bulges in the arterial wall, frequently leading to SAH and disproportionately affecting individuals in their working age. The intricate progression of IA can result in severe and persistent neurological impairments or even fatal consequences. Recognizing the potential debilitating nature of IA, it becomes imperative to investigate the mechanisms dictating its formation, with a primary goal of preventing its progression and rupture. In this pursuit, we conducted an exhaustive exploration of the miRNA-mRNA network and the associated biological pathways linked to IA through rigorous bioinformatics analysis.

**Fig. 10:**
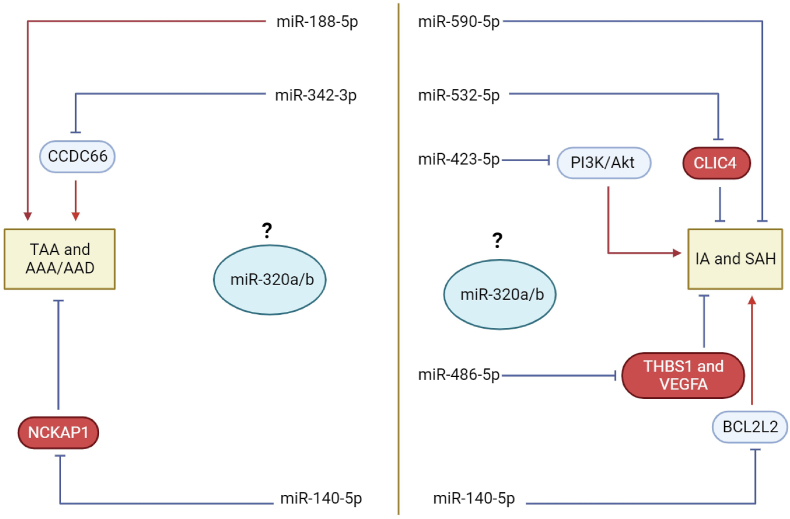
Schematic illustration of the role of the found (in this study) microRNAs (miRNAs) in abdominal aortic aneurysm (AAA), thoracic aortic aneurysm (TAA), and intracranial aneurysm (IA), and acute aortic dissection (AAD) and subarachnoid hemorrhage (SAH).

Aneurysms, characterized by their location, encompass various types, including abdominal aortic aneurysm (AAA), thoracic aortic aneurysm (TAA), and IA. Pathologically, aneurysms manifest features such as inflammatory cell infiltration, remodeling of the extracellular matrix (ECM), compromised arterial wall integrity, and the death of VSMCs. Despite significant progress in understanding aneurysm pathophysiology, the precise molecular mechanisms specific to each type remain elusive [17]. However, we posit that shared miRNAs and signaling pathways exist among different aneurysm types, often overlooked, or underestimated despite anatomical distinctions. Recognizing and elucidating these shared elements holds substantial promise for advancing both fields, particularly in the realm of cellular and molecular-guided therapies. Emphasizing the necessity for cross-disciplinary preclinical studies, this approach stands to enrich our collective comprehension of the intricate landscape of aneurysm pathogenesis (Fig. 10).

In this investigation, DEGs and DEMs were meticulously screened in IA samples relative to normal samples, employing bioinformatic methodologies. It is well-established that miRNAs exert their regulatory influence by binding to the 3′-UTR of target gene mRNAs, thereby either inhibiting the translation process or promoting the degradation of the corresponding mRNA. Within the realm of predicted miRNA-gene pairs, a noteworthy discovery emerged—50 target genes exhibited significant negative correlations with 9 upregulated DEMs, namely hsa-miR-188-5p, hsa-miR-590-5p, hsa-miR-320b, hsa-miR-423-5p, hsa-miR-140-5p, hsa-miR-486-5p, hsa-miR-320a, hsa-miR-342-3p, and hsa-miR-532-5p. This intricate network further unveiled two pivotal miRNAs, hsa-miR-188-5p and hsa-miR-590-5p, along with ten crucial genes (ATP6V1G1, XRCC5, ZFP91, CBFB, KBTBD6, TIMP3, PCBP1, PCBP2, S100A10, and LANCL1). Moreover, previous studies have shown that these genes may involve in vascular inflammation, endothelial dysfunction, oxidative stress, VSMC phenotypic switching, cell apoptosis, etc. Table 4 summarizes the current studies on these 10 genes in vascular pathology [[18], [19], [20], [21], [22], [23], [24], [25], [26], [27], [28], [29], [30], [31], [32], [33]].

**Table 4:** Information of vascular lineage-specific genes.

Cellular unit	Gene	Important find	References
ECs	ATP6V1G1	Promotes the proliferation of ECs and angiogenesis	[18]
ECs	XRCC5	Promotes the proliferation of ECs and angiogenesis	[19]
ECs	ZFP91	The inflammatory response of ECs. Role in the ECs migration and tube formation	[20,21]
ECs	CBFB	Promotes the proliferation of ECs and angiogenesis	[22]
Human PBMC	KBTBD6	Play in innate and adaptive immune responses in vascular inflammation	[23]
ECs, VSMCs and fibroblasts	TIMP3	Was reported to be a potential risk factor of atherosclerosis, aneurysm, and hypertension. Prevents ECM degradation and maintain vascular stability	[[24], [25], [26], [27]]
VSMCs	PCBP1	Role in VSMCs proliferation, migration, apoptosis, and differentiation.	[28]
ECs and VSMCs	PCBP2	Vascular permeability. Oxidative stress	[29]
ECs and macrophage	S100A10	Important regulator of thrombotic and fibrinolytic events in the vasculature. Promotes the proliferation of ECs and angiogenesis. Regulates macrophage differentiation and atherogenesis	[[30], [31], [32]]
ECs	LANCL1	Vascular endothelial dysfunction. Regulate vascular cells response to hypoxia by activating NO generation	[33]

**Abbreviations:** Human PBMC, Peripheral blood mononuclear cells; ECs, Endothelial cells; VSMCs, Vascular smooth muscle cells.

Remarkably, the regulatory pairs within this network showcased the influence of hsa-miR-188-5p on ATP6V1G1/XRCC5/ZFP91/CBFB and hsa-miR-590-5p on TIMP3/PCBP1/PCBP2/S100A10/LANCL1. Previous studies have shed light on the significance of miR-188-5p in AAA progression, demonstrating its upregulation and its impact on elastin degradation, VSMC depletion, and mural angiogenesis inhibition [34]. Similarly, miR-140-5p suppression has been observed in acute aortic dissection (AAD) patients, correlating with upregulated NCKAP1 levels, influencing VSMC proliferation, migration, and invasion [35]. Deng et al., investigated the role of miR-140/BCL2L2 axis on the formation of IAs. The authors found that the expression of miR-140 increased in IA patients [36]. BCL2L2 can significantly promote the proliferation of human brain vascular smooth muscle cells (HBVSMCs) and inhibit apoptosis by negatively regulating miR-140, thus controlling the occurrence of IAs.

The miR-320 family, including miR-320a and miR-320b, collectively hinders VSMC proliferation and migration by targeting Neuropilin 1 (NRP1) [37]. MiR-320b specifically targets apoptosis-resistant E3 ligase 1 (AREL1), thereby influencing apoptosis in human umbilical vein endothelial cells (HUVECs) [38]. Furthermore, the study delves into the context of bicuspid aortic valve (BAV) ascending aortic aneurysm (AsAA), where elevated plasma levels of miR-320a are associated with mid-ascending aortic wall strain [39]. In the realm of circCCDC66, miR-342-3p, and CCDC66, their intricate interplay has been evidenced in human VSMC apoptosis and proliferation in AAA [40]. Understanding the role of miR-532-5p in atherosclerosis progression was elucidated by its regulatory effects on VSMC behaviors [41]. Overexpression of miR-532-5p demonstrated inhibitory effects on VSMC proliferation and migration [42]. Additionally, the impact of miR-532-5p on human brain microvascular endothelial cells (HBMECs) damaged by ox-low-density lipoprotein (ox-LDL) was explored. The findings highlighted the protective role of miR-532-5p in mitigating HBMECs damage induced by ox-LDL, attributed to its down-regulation of intracellular chloride channel 4 (CLIC4) expression [43]. This comprehensive exploration underscores the intricate regulatory networks involving specific miRNAs and their target genes in the context of IA. The multifaceted roles of miRNAs in influencing cellular behaviors and pathways associated with aneurysm progression provide valuable insights for further understanding and potential therapeutic interventions.

SAH stands as a critical neurological condition characterized by a high morbidity and mortality rate, primarily attributed to the rupture of IAs. The exploration of MiRNAs and their regulatory mechanisms, particularly their impact on IA rupture, has garnered significant attention from researchers. MiRNAs emerge as potential therapeutic targets and biomarkers for SAH, holding promise for drug modulation and diagnostic tool development. Notably, various studies have unveiled distinct miRNAs influencing the likelihood of IA rupture.

In an informative analysis by Zhao et al., Weighted Correlation Network Analysis (WGCNA) identified four miRNAs with potential as IA biomarkers, boasting Area Under Curve (AUC) values exceeding 0.75 [44]. Notably, hsa-miR-423-5p exhibited commendable predictive performance. Lopes et al. employed Next-Generation Sequencing (NGS) to scrutinize miRNA expression in peripheral blood samples from SAH patients, identifying eight DEMs, of which miR-486-5p, displaying downregulation, correlated with poor neurological admission status [45]. Furthermore, studies suggest that VSMC-secreted exosomes transfer miR-486 into endothelial cells (ECs), inhibiting their migratory activities in physiological conditions [46]. Zheng et al.'s microarray analysis delved into plasma miRNA profiles in SAH patients, revealing the significant downregulation of miR-590-5p, indicating its association with IA rupture [47].

Pathway enrichment analysis of target genes implicated inflammation, VSMC proliferation, and cell adhesion as potential contributors to disease occurrence. Intriguingly, the present study identified an increase in the expression of 9 miRNAs, contrasting with the results of studies. Additionally, observed discrepancies between our findings and those investigating aortic aneurysms underscore the intricate and multifaceted nature of aneurysms, suggesting avenues for further exploration. This study not only sheds light on the complex landscape of aneurysms but also proposes novel goals and strategies for advancing the understanding and study of SAH.

Our findings highlight the significance of the AMPK signaling pathway in IA, a conclusion consistent with prior research [48,49]. Li et al. conducted a study demonstrating that metformin exerts a protective effect against IA formation and rupture by impeding VSMC phenotype switching, as well as inhibiting VSMC proliferation, migration, and apoptosis in vivo [48]. Notably, the study revealed AMPK pathway activation under various metformin doses, underscoring its role in restraining VSMC activities. In a related study, Sun et al. observed that AMPK activation mitigated the pro-inflammatory effects of miR-323a-3p in the human ECs line EA.hy926 [49]. Their findings suggested that targeting the AMPK signaling pathway through miR-323a-3p could hold promise for future anti-inflammatory treatments for IAs. These results not only affirm the importance of the AMPK pathway in IA but also offer potential therapeutic avenues by modulating this pathway to counteract the inflammatory processes associated with IA development.

To gain deeper insights into the regulatory mechanisms of target genes within DEMs, our study identified potential TFs, with SP1, SP4, KLF7, and EGR1 emerging as the most prevalent. In a recent study, SP1 was confirmed to mediate the impact of miR-335-5p on the phenotypic switching of VSMCs in AAD, exhibiting a pro-apoptotic influence by repressing the expression of p21WAF1/Cip1 at the transcriptional, mRNA, and protein levels [50,51]. Additionally, SP1 and SP4 have been recognized as key TFs involved in the regulation of vascular endothelial growth factor (VEGF) production by binding to specific sites in the VEGF promoter [52,53]. KLF7's involvement in various cardiovascular diseases is well-documented. It inhibits pulmonary arterial smooth muscle cells' (PASMCs) proliferation and migration through the p21 pathway in vitro and in vivo [54]. Studies have shown that KLF7 expression decreases in oxidized low-density lipoprotein (ox-LDL)-induced HUVECs and inhibiting KLF7 reverses the inhibition effect of miR-301a-3p, promoting inflammation, apoptosis, and oxidative stress in ox-LDL-induced HUVECs [55]. EGR1, identified as a transcription factor activated by vascular injury, has been implicated in the pathogenesis of various vascular diseases, including AAA, TAA, atherosclerosis, myocardial ischemia/reperfusion injury, hypertension, and pathological angiogenesis [[56], [57], [58], [59], [60], [61], [62]]. This underscores the potential of EGR1 regulation as an exploitable target in IAs. Subsequent GO and pathway enrichment analyses of target mRNAs or PPI networks revealed enrichments in cellular characteristics, the establishment of protein localization, the mitochondrion, and enzyme binding. These findings align with prior investigations that have identified apoptosis, ECs proliferation, VSMCs, and immune cell infiltration as key contributors to IA [63,64]. Our results further substantiate the regulatory role of miRNAs in these fundamental pathophysiological processes, underscoring the pivotal role of miRNAs in the context of IAs.

Despite the results, this study has some limitations. The lack of complete human tissues reflecting the characteristics of the disease limits bioinformatics research in the field of IA. It is known that the expression of miRNAs or genes can be influenced by many factors, including comorbidities or types of tissue samples, which are definitely important for obtaining more reliable results. For example, the GSE66240 database contained healthy STA tissue from IA patients as a control group. Besides, the source of blood and cerebrospinal fluid (CSF) samples will more accurately explain the physiological and pathological process of IA formation and progression. This is one of the limitations of the present study. In addition, there is a need to study with other databases in patients with SAH. Further the sample size of datasets chosen is not huge enough. Also, in our study there is no the AUC analysis which represented the ability of miRNAs to distinguish IA samples from normal samples, and AUC >0.75 could be set as the criteria for screening DEMs analysis It is primarily this bioinformatics analysis used for potential pathogenesis analysis. These DEMs and DEGs may be key nodes in the occurrence and development of IA. This provides new insights into the pathogenesis of IA. However, this study still needs to be confirmed by further preclinical and clinical experiments for finding new diagnostic and therapeutic tools.

## 5 Conclusion

In this investigation, we have successfully identified pivotal miRNAs, their target mRNAs, and crucial pathways associated with IA. Particularly noteworthy are hsa-miR-188-5p and hsa-miR-590-5p, among a total of 9 upregulated miRNAs, which exhibited substantial increases in IA. This upregulation correlated with the downregulation of their target genes, including ATP6V1G1, XRCC5, ZFP91, CBFB, KBTBD6, TIMP3, PCBP1, PCBP2, S100A10, and LANCL1. These findings significantly contribute to a more comprehensive understanding of the potential mechanisms underpinning IA formation and progression, thereby offering novel insights for both diagnostic approaches and therapeutic interventions related to IA. It is imperative, however, to recognize the intricate nature of the miRNA-mRNA interaction network, emphasizing the need for further in vitro and in vivo investigations to validate our results and assess their potential clinical applicability in the context of IA.

## Funding

This work was supported by the Key Research and Development Projects in Heilongjiang Province (2022ZX06C03) and the Bashkir State Medical University Strategic Academic Leadership Program (PRIORITY-2030)

## Patient consent for publication

Not applicable.

## Ethics approval and consent to participate

Not applicable.

## CRediT authorship contribution statement

**Dongxiao Xu:** Writing – review & editing, Writing – original draft, Project administration, Conceptualization. **Ilgiz Gareev:** Writing – original draft, Project administration, Conceptualization. **Ozal Beylerli:** Writing – review & editing, Visualization, Validation, Investigation. **Valentin Pavlov:** Resources, Data curation. **Huang Le:** Formal analysis, Data curation. **Huaizhang Shi:** Supervision, Resources, Funding acquisition, Data curation.

## Declaration of competing interest

Ozal Beylerli is an editorial board member for Non-coding RNA Research and was not involved in the editorial review or the decision to publish this article. All authors declare that there are no competing interests.
